# Adult Vaccinations Today—Innovations and Challenges for the Coming Years

**DOI:** 10.3390/vaccines13060583

**Published:** 2025-05-29

**Authors:** Helena C. Maltezou, Dimitrios C. Cassimos, Nikolaos V. Sipsas, Snezana Medic

**Affiliations:** 1 Directorate for Research, Studies, and Documentation, National Public Health Organization, 15123 Athens, Greece; 2 Pediatric Department, Democritus University of Thrace, 68100 Alexandroupolis, Greece; dkasimos@med.duth.gr; 3 Pathophysiology Department, Medical School, National and Kapodistrian University of Athens, 11527 Athens, Greece; nsipsas@med.uoa.gr; 4 Department of Epidemiology, Faculty of Medicine, University of Novi Sad, 21000 Novi Sad, Serbia; snezana.medic@mf.uns.ac.rs; 5 Center for Disease Control and Prevention, Institute of Public Health of Vojvodina, 21000 Novi Sad, Serbia

**Keywords:** adults, comorbidities, vaccinations, vaccination programs

## Abstract

Routine pediatric vaccinations have resulted in dramatic declines in the incidence and complications of several vaccine-preventable diseases (VPDs) over the past fifty years. At the same time, the prolongation of life expectancy and the large number of adults living with chronic medical conditions changed the demographic profile and, accordingly, the healthcare needs. The recognition of the burden and effects of several VPDs in adults and in particular risk groups and the comprehension of the age-dependent deterioration of immune responses have driven the extension of routine vaccination programs beyond adolescence. In addition, several VPDs emerged or re-emerged over the past decades, and new vaccines have been developed or are under clinical assessment. Currently, vaccination programs in many countries include vaccinations for adults, aiming to expand and strengthen protection throughout the lifespan and promote healthy aging. Moreover, there are needs for new or more effective vaccines against common or emerging pathogens and public health threats, including chronic diseases. This article reviews the current status of several adult vaccinations and discusses challenges for adult vaccinations, including new vaccines, emerging or re-emerging VPDs, and strategies to overcome low vaccination rates.

## 1. Introduction

Childhood vaccinations have been a major public health achievement, accounting for a 40% reduction in infantile mortality in the past fifty years [1,2]. Nevertheless, our knowledge about the effects of several vaccine-preventable diseases (VPDs) on adults and particularly on specific groups (e.g., pregnant women) has expanded significantly in the past decades, along with the prolongation of life expectancy and the increasing number of people living with chronic medical conditions [3]. Currently, people ≥ 65 years old account for 10.3% of the global population, and according to United Nations projections, this number is expected to double by 2074 [4]. The comprehension of immunosenescence, characterized by the age-dependent deterioration of the immune system’s capacity to respond to infections or vaccination, and the recognition of the need for vaccine boosters to sustain protection throughout the lifespan, have shaped modern vaccination programs [5,6]. In addition, in the past three decades several VPDs emerged [e.g., coronavirus disease 2019 (COVID-19)] or re-emerged (e.g., pertussis), and new vaccines were developed [e.g., respiratory syncytial virus (RSV) vaccines] [7,8,9,10]. Currently, vaccines constitute a significant preventive measure during adulthood, and in many countries, childhood vaccination programs have evolved into vaccination programs for all ages and groups [1,5,11,12]. This article reviews recent advances in vaccinations and vaccination strategies for adults and discusses vaccination challenges for the coming years, including the emergence or re-emergence of several VPDs. Strategies to overcome low vaccination rates among adults are also discussed.

## 2. Enhanced Influenza Vaccines for Older Adults

Influenza has a significant impact, particularly on older adults. Surveillance data from the United States (US) over nine seasons (2010–2019) estimated a 12.2% case fatality rate among patients ≥ 85 years hospitalized with influenza compared with 0.5% in hospitalized children aged 0–4 years [13]. This study also captured the significant post-discharge mortality among patients hospitalized with influenza (ranging by season from 35.9% to 54.5% of all deaths that occurred either in hospital or by 30 days post-discharge), which increased considerably with increasing age (from 12.5% of all deaths among patients aged 0–4 years to 59.7% of all deaths among patients ≥ 85 years) [13]. Overall, patients ≥ 65 years accounted for 76% of all deaths that occurred among hospitalized patients with influenza, either in hospital or by 30 days post-discharge [13].

To address the limited effectiveness of standard-dose (SD) influenza vaccines in older adults, high-dose (HD), adjuvanted, and recombinant influenza vaccines (collectively called enhanced influenza vaccines) were developed [14]. A meta-analysis of 32 studies between 1990 and 2023 with 71.4 million vaccinated persons found that enhanced influenza vaccines were more effective (by 11–18%) against influenza-related hospitalizations in adults ≥ 65 years compared with SD influenza vaccines [15]. Currently, HD, adjuvanted, and recombinant influenza vaccines are recommended for adults ≥ 65 years by several public health bodies; however, evidence comparing HD, adjuvated, and recombinant influenza vaccines is limited [15,16]. The receipt of two influenza vaccinations annually has also been considered as a strategy to overcome the waning effectiveness of influenza vaccines over time, particularly for older adults [17]. Real-world data about the effectiveness, potential adverse effects, and additional costs of this strategy are needed [17]. Lastly, there is significant evidence that influenza vaccination offers cardiovascular benefits beyond those associated with the direct protection against influenza [18]. A meta-analysis of 15 studies with 745 thousand patients has found that the influenza vaccination of patients with cardiovascular disease is associated with lower rates of all-cause mortality, cardiovascular death, and stroke [19].

## 3. Herpes Zoster Vaccine

Herpes zoster (HZ) is caused by the reactivation of the varicella-zoster virus (VZV), which after the primary infection of varicella (chickenpox) remains in a latent phase in the nervous system. Immunosenescence and T-cell immunosuppression contribute to the reactivation of the VZV and the development of HZ. The incidence of HZ increases considerably with increasing age, mainly after the age of 50 years, in females, and in the presence of comorbidities, particularly immunosuppression [20,21]. Postherpetic neuralgia is a long-lasting, painful complication of HZ associated with loss of quality of life. Other HZ complications include retinitis, keratitis, vasculitis, myelitis, stroke, and Guillain–Barré syndrome. Due to viremia, HZ in immunocompromised patients may involve several systems with pulmonary, hepatic, and central nervous system manifestations and recurrent HZ [22].

A live attenuated HZ vaccine which contains 14 times the amount of the same virus strain used in the varicella vaccine for children, was licensed in 2006 [14]. The live attenuated HZ vaccine is administered only to immunocompetent adults. Real-world studies have shown declining vaccine effectiveness with increasing age and post-vaccination time [14,23]. Currently, the live attenuated HZ vaccine is available in few countries.

In 2017, an adjuvanted recombinant HZ vaccine was licensed. This vaccine contains a recombinant glycoprotein E of the virus [14]. Preliminary studies have shown substantially higher immunogenicity (both antigen-specific T-cell responses and antibody responses) with the AS01-adjuvanted formulation, particularly in older adults, compared with non-adjuvanted formulations [14]. An overall 81% decrease in the risk ratio of HZ infection in severely immunocompromised patients across all age groups has been estimated in a meta-analysis of randomized clinical trials with the recombinant HZ vaccine [24]. In addition, vaccination with the recombinant HZ vaccine significantly increases cellular and humoral immunity one month after the second dose [24]. Real-world data indicate that efficacy is sustained for almost ten years after vaccination [14]. The recombinant HZ vaccine is the first vaccine that has been shown to induce a robust cellular response in older adults [14]. Currently, the recombinant HZ vaccine is the preferred HZ vaccine for older adults and adults with chronic medical conditions in several countries [14]. Yet, despite the rapidly ageing human population in many countries, the HZ vaccine uptake rates remain low worldwide [25].

## 4. COVID-19 Vaccines

The extremely rapid development of lifesaving COVID-19 vaccines in parallel with the first pandemic waves was unprecedented in the history of medicine. By December 2020, several different vaccine platforms targeting SARS-CoV-2 were developed [26]. Previous vaccine research along with the possibility of rapid vaccine approval in emergency situations and the availability of funding contributed to this success [27,28]. The two most widely used COVID-19 vaccines were messenger ribonucleic acid (nRNA) vaccines which both used nucleoside-modified mRNA encoding a variant of the spike protein found on the surface of SARS-CoV-2 [14]. Both mRNA vaccines induced strong T-cell and B-cell responses, though they were lower in older adults [14]. Nevertheless, real-world effectiveness was high, particularly against severe disease [14]. It is estimated that 19.8 million deaths from COVID-19 were prevented during the first year of COVID-19 vaccination alone [26]. Since their first approval in late 2020, the composition of COVID-19 vaccines has changed several times [29]. In 2022, variant-adapted vaccines began to be approved for use as boosters [30]. In 2023, adapted vaccines that protect against a strain belonging to the XBB Omicron family of SARS-CoV-2 were approved [30,31,32]. In April 2024, monovalent adapted vaccines targeting the JN.1 Omicron subvariant family were recommended by the World Health Organization (WHO) and several public health authorities globally [29,33].

Although recommendations for vaccination against COVID-19 exist in most countries, there are significant variations in terms of vaccination schedules, vaccine types, and priority high-risk groups [12,34,35,36]. Most recommendations for COVID-19 vaccination prioritize people ≥ 60 years, healthcare personnel, patients with comorbidities, pregnant women, residents of long-term care facilities, and close contacts of at-risk patients [30,36]. To overcome vaccination barriers and raise convenience for vaccinees, COVID-19 vaccination is recommended simultaneously with influenza vaccination [35,36,37]. Monitoring of adverse events following the administration of approved COVID-19 vaccines has shown that the benefits of vaccination outweigh the potential risks [38,39]. In addition, there is significant evidence that COVID-19 vaccination significantly reduces the risk of post-COVID conditions, regardless of vaccine type, number of vaccine doses, circulating SARS-CoV-2 variant, and severity of acute infection [40]. Adjustments to vaccination recommendations will be necessary in terms of updating risk and age groups, number of additional doses, and development of next-generation vaccines against the dominant circulating SARS-CoV-2 variant [36,41].

## 5. Pneumococcal Vaccines

Pneumococcal disease remains a significant cause of morbidity, mortality, and hospitalization costs in adults, often in association with high proportions of antibiotic-resistant *Streptococcus pneumoniae* strains [42,43,44]. A study from 2016 to 2022 in Spain estimated an overall hospitalization rate for pneumococcal disease of 108.9 hospitalizations per 100,000 population, which increased notably with age (up to 748 hospitalizations per 100,000 population ≥ 90 years) [45]. Overall, hospitalizations for pneumococcal disease contributed EUR 384 million annually to the costs of the Spanish healthcare system [45]. Patients with diabetes mellitus and immunocompromised patients, particularly HIV patients, patients with solid organ transplants, and patients with autologous or allogeneic stem cell transplants are at even higher risk for invasive pneumococcal disease (IPD) [46,47]. A meta-analysis of 26 studies of patients aged 15–64 years old with IPD found an overall 30-day mortality or in-hospital mortality rate of 20.8% (95% CI: 17.5–24%); factors associated with increased mortality rate were increased age, residence in a nursing home, nosocomial infection, septic shock, underlying chronic diseases, solid organ tumors, immunosuppression, and alcohol abuse [48].

Adverse cardiovascular events occur frequently in patients with IPD. A retrospective study of IPD cases diagnosed from 2012 to 2019 in Colombia found an overall 23% prevalence of major cardiovascular events among IPD patients [49]. In this study, patients with serotype 3 or 9n infections were significantly more likely to develop adverse cardiovascular events, as were those with bacteremia or previous cardiovascular risk factors [49]. A cohort-based study of 914 patients ≥ 45 years old with IPD hospitalized in the Netherlands from 2012 to 2020 also found an association between the 7 F serotype and acute coronary syndrome and between the 22F serotype and stroke [50].

The 23-valent pneumococcal polysaccharide vaccine (PPSV23) has been used for more than four decades in older adults and individuals with high-risk medical conditions; however, it is ineffective in young children and does not induce long-term protection [43]. To address the needs of young children, the 7-valent pneumococcal conjugate vaccine (PCV) was launched in 2000, followed by the 10-valent and the 13-valent PCVs a decade later. PCVs induce more robust immune responses across all age groups than PPSV23. However, the introduction of each PCV was initially associated with a reduction in IPD incidence, followed by a steady rise in IPD incidence along with the emergence of non-vaccine serotypes [43]. For instance, following the replacement of the 7-valent PCV in routine vaccination programs for infants with the 10-valent and the 13-valent PCVs, the incidence of IPD decreased across all age groups, including older adults, due to herd immunity; nevertheless, the non-13-valent vaccine serotypes increased notably alongside another increase in the non-PCV IPD incidence in multiple countries, limiting the overall benefits of pediatric vaccination programs [51,52,53]. Moreover, infections with serotype 3, which is contained in the 13-valent PCV, have emerged among older adults, along with infections with 19A, 19F, 8, 22F, and 9N serotypes [43]. Overall, the distribution of serotypes involved in pneumococcal infection and disease vary by region, time, and setting.

The introduction of the 15-valent and the 20-valent PCVs in 2021 provided additional protection through additional serotypes (up to one-third and two-thirds of cases in older adults in 10 European countries, respectively) [52]. It has been estimated that the use of the 15-valent and the 20-valent PCVs could prevent up to 20.2–27.4 additional lower respiratory tract infections (LRTIs), 2.2–3.5 additional LRTI-related hospitalizations, and 0.6–1.0 additional excess LRTI-related deaths per 10,000 person-years among adults ≥ 18 years in the US [54]. A recently published systematic review of 26 cost-effectiveness studies in adults, found that in most studies the 20-valent PCV administered alone was cost-saving or cost-effective compared with other adult pneumococcal vaccination strategies, including vaccination with the PPSV23, while protection was assumed to last 10–20 years [55]. In 2024, a 21-valent PCV was licensed in the US for use in adults ≥ 18 years [43]. This vaccine contains eight serotypes unique to the adult population, and according to surveillance data and model estimates, it is expected to provide additional coverage against IPD isolates compared to the 15-valent and the 20-valent PCVs and the PPSV23, including resistant isolates, particularly in adults ≥ 65 years [56]. Both the 20-valent and the 21-valent PCVs demonstrated a good safety and tolerability profile and immunogenicity in adults against the respective vaccine-containing serotypes [57,58,59]. The US CDC recommends either the 20-valent or the 21-valent PCV or the 15-valent PCV followed by the PPSV23 for adults ≥ 50 years and for adults aged 19–49 years with particular medical conditions [43]. Coadministration of PCVs with COVID-19 or influenza vaccines is safe and well tolerated and is expected to raise vaccination compliance [60,61].

## 6. RSV Vaccines

During the winter season, RSV is a main cause of LRTIs, which are subsequently associated with significant morbidity and mortality worldwide. Premature young infants, older adults, residents of nursing homes, and individuals with comorbidities, e.g., chronic pulmonary disease, chronic cardiovascular disease, and immunosuppression (e.g., solid organ transplant recipients and hematopoietic cell transplant recipients), are at higher risk for RSV-associated hospitalization, severe clinical course, and unfavorable outcome [62,63,64,65,66]. A population-based study conducted in Spain from 2016–2017 to 2019–2020 estimated a mean annual rate of RSV hospitalizations of 84.7 per 100,000 adults ≥ 60 years [67]. Risk factors for RSV hospitalization were advanced age, nursing home residence, functional dependence, and specific comorbidities [67]. Particularly in infants, RSV disease is often associated with asthma and reduced respiratory function later in life [62]. A meta-analysis of 21 studies conducted from 2000 to 2021 in the US, France, Germany, Spain, Italy, Japan, and the United Kingdom (UK), estimated that in 2025 it is expected that RSV will be responsible for 5.7 million infections and 510,000 hospitalizations among adults ≥ 60 years, while it is expected that 37,000 adults ≥ 60 years will die in these countries only [68]. Lastly, a model-based study using data from 2015 to 2018 estimated that a mean of 640,000 antibiotic prescriptions in general practice settings in England every year are attributed to RSV, while adults ≥ 75 years old accounted for one in every four antibiotic prescriptions for RSV [69].

Currently there are three RSV vaccines licensed by the US Food and Drug Administration and the European Medicines Agency [10]. The prefusion F protein of RSV was selected for the development of the currently licensed RSV vaccines because of considerable stability through time, for both RSV-A and RSV-B subtypes, and because it has highly immunogenic sites [70]. The first two licensed RSV vaccines, the RSVPreF3, which contains the AS01 adjuvant, and the bivalent RSVpreF, are subunit vaccines and were developed with DNA technology which has been advantageous regarding safety and limited side effects. The third vaccine, an mRNA vaccine called mRNA-1345 vaccine, induces the production of specific antigens by the host cells, triggering an immune response against these RSV antigens [10,71,72]. The three RSV vaccines are approved for older adults (with different age cut-offs) and adults with high-risk comorbidities, while the RSVpreF vaccine is also licensed for pregnant women of 32–36 weeks gestation to protect young infants through transplacental transfer of vaccine-derived RSV-specific IgG antibodies [10]. Currently, there are several RSV vaccines of various technologies under development or in clinical trials [63,70].

All three licensed RSV vaccines demonstrated very good effectiveness against LRTI-associated emergency department (ED) visits and hospitalizations and laboratory-confirmed RSV infections and associated ED visits and hospitalizations; the two subunit vaccines also show effectiveness that persists over the second RSV season [66,70,73,74,75]. Preliminary data from the 2024–2025 season in the UK indicate a 30% [95% confidence interval (CI): 18–40%] reduction in hospitalizations for RSV among adults aged 75–79 years with a 47.4% vaccine coverage with the bivalent RSV vaccine by 6 January 2025 [76]. These real-world data indicate a clear effect of RSV vaccination on RSV-related hospitalizations [76]. Studies also have shown that post-vaccination neutralizing titers before the start of the second RSV season remained substantially higher than baseline pre-vaccination titers [73]. However, a recent study among 38 vaccinated immunocompromised patients (21 with the RSVPreF3 vaccine, 14 with the RSVpreF vaccine, and 3 in whom the vaccine brand was unknown) found that the studied patients had a mean of a 4.21-fold rise of pre-F IgG antibodies four weeks post-vaccination, while overall only 23 (61%) of them had evidence of seroconversion [77]. Although the number of studied patients was small, these findings indicate that immunocompromised patients may benefit from a two-dose vaccine schedule [77].

## 7. Pertussis Vaccines

Vaccinations of young children with whole-cell pertussis vaccines were widely implemented from the middle of the 20th century, leading to an enormous decrease in the associated morbidity and mortality in the following decades [1,78]. Nevertheless, due to adverse events, the whole-cell pertussis vaccines were replaced by acellular pertussis vaccines in the early 1990s in many countries [7]. In the past three decades, pertussis has re-emerged in several countries with high vaccination rates among young children, along with an increase in cases among adolescents and adults [7,8,79]. The re-emergence of pertussis and the swiftness of the peak from childhood to adolescence and adulthood is explained by the waning protection of acellular pertussis vaccines. Other reasons include better diagnosis and surveillance of pertussis cases and possibly changes in circulating *Bordetella pertussis* strains [7,8].

In adolescents and adults, pertussis usually manifests with atypical symptoms and therefore diagnosis may be delayed [7,80]. A large US study found that young children under the age of two years with pertussis had a mean diagnostic delay of 5.6 days compared to a mean of 13.8 days among adults with pertussis [81]. Overall, pertussis is a low-perception VPD for adults by physicians, which also explains the underdiagnosis of pertussis in adulthood [82]. *B. pertussis* infection appears to be associated with an increased likelihood of developing asthma and chronic obstructive pulmonary disease (COPD) [83]. Conversely, patients with asthma or COPD are at increased risk for severe pertussis in terms of duration and severity of symptoms and risk of hospitalization, as well as for asthma or COPD exacerbations, which are attributed to the intense inflammatory pathophysiology of pertussis [83]. Moreover, *B. pertussis* infection in older adults and adults with co-morbidities is also associated with serious morbidity, occasional mortality, and considerable healthcare costs [80,83,84]. Pertussis in adolescents and adults is also of concern because of transmission of infection to newborns and young infants [7]. Adult-type acellular pertussis component vaccines have been in use particularly in adolescents and adults for more than three decades to directly protect them but also to indirectly protect young infants from transmission of *B. pertussis* [5,7,85]. Surveillance data and cost-effectiveness studies indicate the benefits of booster vaccine doses in adolescents and adults on hospitalization rates for pertussis among infants < 2 months [86,87]. Currently, several countries recommend booster doses with adult-type vaccines every ten years throughout life [5].

Pertussis vaccination during pregnancy is an increasingly used vaccination strategy aiming to protect young infants against severe pertussis through transplacental transfer of vaccine-derived IgG antibodies [11]. The timing of pertussis vaccination is a key determinant for conferring protection to young infants, given the gradual waning of antibodies over time [11]. Most countries recommend pertussis vaccination during the third trimester [11].

The epidemiology of pertussis in the past thirty years has been largely shaped by the gap of lifelong protection following *B. pertussis* infection and/or vaccination with the current pertussis vaccines. In addition, there are significant variations in pertussis vaccination programs across countries in terms of vaccines used, number of doses, recommended ages, and recommendations for booster doses in children, adolescents, adults, and pregnant women [5,11,79,85]. More efficacious pertussis vaccines but of similar excellent safety are needed particularly for pregnant women, to avoid vaccinations in each pregnancy [79].

## 8. New Vaccination Strategies: Cocooning Strategy

Young infants with influenza are at increased risk for serious illness, use of healthcare services, hospitalizations, and death [88,89]. Similarly, most severe cases and hospitalizations due to pertussis concern young infants under the age of six months, while this age group accounts for more than 90% of pertussis deaths across all age groups [7,8,90]. Cocooning vaccination strategies (or nest immunity), defined as vaccination of close contacts (family members and carers) of young infants, have been increasingly used in the past two decades to address the disproportionate morbidity and mortality risk that specific VPDs carry for young infants until they later reach the necessary age for full vaccination. Real-world data show that postpartum maternal influenza vaccination confers significant effectiveness against influenza-like illness, healthcare seeking, and prescription of antibiotics [91]. Evidence shows that the availability of vaccinations during obstetric admission before discharge from the maternity hospital is associated with increased vaccine uptake rates [91,92]. Nevertheless, maternal vaccination during pregnancy against pertussis and influenza offers more benefits, mainly the protection of the pregnant woman but also the protection of young infants during the first months of life [7,11,79,87,93]. Maternal vaccination during pregnancy also provides “cocooning” protection, since mothers represent the most frequent source of pertussis and influenza infection for their babies [11,91,93].

## 9. Meningococcal B Vaccination to Protect Against Gonorrhea

Gonorrhea, a sexually transmitted disease (STD) with serious complications, is reemerging globally along with the emergence of multidrug-resistant strains. According to the WHO, in 2020 there were more than 80 million new infections worldwide [94]. The number of gonorrhea cases is likely underestimated since many infections are asymptomatic and therefore escape diagnosis.

Real-world studies have shown that the use of outer membrane vesicle (OMV)-based vaccines against *Neisseria meningitidis* serogroup B has been associated with a decrease in gonorrhea cases. Massive vaccinations of children, adolescents, and young adults with a bivalent, OMV-based vaccine against a hypervirulent strain B and serogroup C in Cuba in 1989 and 1990, resulted in a dramatic reduction in gonorrhea cases in adolescents and young adults in this country, while this was not the case with other STDs [95]. Surveillance data also indicated a decrease in the incidence of gonorrhea among unvaccinated individuals, which points to herd protection [95]. Reductions in the incidence of gonorrhea were also documented in New Zealand and Norway after the wide use of epidemic-specific OMV-based meningococcal vaccines in these countries [96]. It has been estimated that the efficacy of OMV-based meningococcal serogroup B vaccines against gonorrhea in adolescents and young adults is approximately 40% [97]. The protection conferred by OMV-based meningococcal serogroup B vaccines against gonorrhea is driven by bactericidal IgG antibodies against epitopes common in *N. meningitidis* and *N. gonorrheae* [97].

In response to increasing numbers of gonorrhea cases in the UK in the past few years, including ceftriaxone-resistant *N. gonorrhoeae* cases [98], in November 2023 the Joint Scientific Committee on Vaccination and Immunisation in the UK advised off-label vaccinations with the 4CMenB vaccine of individuals at high-risk of gonorrhea [97]. Vaccinations with the 4CMenB vaccine are delivered at sexual health services and are expected to confer significant protection against gonorrhea in high-risk individuals. There are several unanswered questions, particularly regarding the duration of protection against gonorrhea and the possibility of protection against asymptomatic infection and transmission of infection [97]. This off-label vaccination program represents an expansion of current pediatric vaccination recommendations against invasive meningococcal disease serogroup B to protect high-risk adult groups against gonorrhea.

## 10. Current Epidemiological Situation of VPDs Globally

Over the past five decades, vaccines have saved more than 150 million lives [99]. Yet cases and epidemics of VPDs, such as measles, are on the rise worldwide. As a result of the decline in measles-containing vaccine (MCV) coverage during the COVID-19 pandemic, the estimated number of measles cases increased to 10.3 million in 2023, a 20% increase compared to 2022 [100]. In the European Union/European Economic Area (EU/EEA), over 35,000 people had measles in 2024, ten times more than in 2023 [101]. The majority, as many as 86% of these cases, were unvaccinated against measles. Immunization disruptions during the COVID-19 pandemic have resulted in large measles outbreaks in Africa and the Indian subcontinent [102]. The increase in measles cases worldwide is the result of an accumulation of susceptible individuals, primarily adults, as confirmed by serosurveys, even before the COVID-19 pandemic [103,104,105]. The prevention of measles virus transmission requires vaccination coverage of at least 95% with two doses of the MCV vaccine. A strategy of vaccinating susceptible young and middle-aged adults could be successful in interrupting measles virus transmission in the future [104,105].

At the end of 2024, five EU/EEA countries reported the detection of circulating vaccine-derived poliovirus type 2 (cVDPV2) in wastewater samples [103]. In parallel, it is estimated that around 600,000 children aged 12 to 23 months in the EU/EEA did not receive a primary series of polio vaccines in 2022 and 2023 [103]. A tenfold increase in pertussis cases in 2023–24 compared to the previous two years has been reported in several EU/EEA countries [106]. The most effective prevention of pertussis is to ensure timely administration of the recommended pertussis-containing vaccines.

From the beginning of 2022 to April 2025, a total of 536 diphtheria cases caused by *Corynobacterium diphtheriae* were reported in the EU/EEA. A diphtheria outbreak was reported among migrants in Germany at the end of April 2025. Timely vaccination in childhood along with continued vigilance regarding diphtheria is crucial, especially in medical facilities serving high-risk populations [107].

Overall, an estimated 14.5 million children missed all of their routine doses of vaccines in 2023 (zero-dose children) [108]. Immunization successes today are threatened by humanitarian crises and reduced funding. Addressing the problem of inadequate vaccination coverage in adults requires understanding the causes of growing hesitancy to vaccinate, driven by concerns about vaccine safety and efficacy, lack of need for vaccination due to low prevalence or severity of disease, difficulties in accessing vaccines, socioeconomic inequalities, and declining trust in government and scientific institutions, fueled by the COVID-19 pandemic and the accompanying infodemic [99,100].

In adults, especially those over 50 years of age, VPDs such as pneumococcal disease, influenza, pertussis, and herpes zoster pose a significant burden [109]. Over the past three decades, many countries have expanded their vaccination schedules with many life-saving recommended vaccines as well as booster doses at different stages of life [106]. The need for adults to be up to date and fully vaccinated with routine pediatric vaccines (especially MCV) is becoming a priority in light of the resurgence of measles, pertussis, and other VPDs. Therefore, adherence to vaccination schedules, regular verification of vaccination status of individuals of all ages, and administration of age-appropriate versions of missed or delayed childhood vaccines become an important strategy to close the immunity gaps in the population.

In the post-pandemic period, global tourism has returned to pre-pandemic levels, accompanied by an increase in the incidence of VPDs relevant to travelers [110]. The frequent travel-related VPD risk is highest for influenza, COVID-19, dengue, and yellow fever [110]. There is also a significant risk of travelers contracting hepatitis A, typhus, rabies, and Mpox. During pre-travel counseling, it is necessary to check vaccination records for routine childhood vaccines (tetanus, measles, whooping cough, hepatitis B, etc.) and, if necessary, to get vaccinated. The incidence of VPDs in the destination country, the cumulative exposure of the traveler, the type and level of risk associated with the traveler’s upcoming activities, and his/her financial capabilities must be taken into account when making a decision about vaccination. Ultimately, vaccination is a discretionary but important decision that the prospective traveler should make after consulting with a travel health professional. More details on vaccination for international travelers are beyond the scope of this review.

## 11. An Overview of Vaccination Programs for Adults

In the last two decades, adult national immunization programs (NIPs) have expanded significantly, particularly in high-income countries. This trend is driven by the recognition of the burden of VPDs in adulthood, the progressive ageing of the population, the increasing proportion of adults with comorbidities, and the development of new and updated vaccines [5]. A lifelong approach coupled with prioritizing vaccination of high-risk groups, including pregnant women and older adults, appears to be an effective strategy for reducing the burden of severe VPDs, complications, and deaths [111]. A review of adult vaccination policies in Europe, the US, and Australia revealed significant differences in the number of targeted VPDs, age for vaccination, vaccination schedules, vaccine types, funding mechanisms, and vaccination implementation frameworks (voluntary of mandatory) [5,12,111].

In Europe, vaccination policies for adults are inconsistent despite the globally set targets for the control and elimination of VPDs by the WHO and other organizations, particularly in terms of legal support for the implementation of these programs [111]. Countries in the WHO European Region have adopted the European Immunization Agenda 2030, which defines a strategy to ensure lifelong protection against VPDs [112]. According to the available data, all 30 EU/EEA countries have vaccination programs for adults (referring to those ≥18 years) but with substantial differences in terms of vaccination recommendations and implementation status [5,12]. According to the European Center for Disease Prevention and Control [12], the majority of European NIPs for adults target influenza (30 countries), COVID-19 (29 countries), pertussis (26 countries), and pneumococcal infections (23 countries) (Table 1). Conversely, a negligible number of countries generally recommend adult vaccination against hepatitis B and RSV disease (four countries each), hepatitis A (three countries), and *Haemophilus influenzae* type b (Hib) (two countries). The average number of VPDs included in the NIPs of 30 European countries is 8 (ranging from 2 in Malta to 17 in Spain) (Table 1).

In the US, the Advisory Committee on Immunization Practices (ACIP) currently recommends vaccination against 18 VPDs, including Hib, meningococcal serogroups A, C, W, Y, meningococcal serogroup B, measles, mumps, rubella, pneumococcus, tetanus, diphtheria, pertussis, varicella, hepatitis A, hepatitis B, RSV, human papillomavirus, influenza, COVID-19, and HZ for all or for specific age groups of adults aged ≥19 years, including catch-up vaccination for missed childhood vaccines (Table 1) [1]. In addition, ACIP recommends vaccination against influenza at any time during pregnancy and the tetanus, diphtheria, and pertussis vaccination in the third trimester of each pregnancy [1]. Adult vaccination in the US also includes vaccination of high-risk groups against 12 VPDs including influenza, RSV, tetanus, diphtheria, pertussis, HZ, pneumococcal and meningococcal infections, hepatitis A, hepatitis B, Hib, and Mpox [113,114].

The national recommendations for vaccination issued by the Australian Technical Advisory Group on Immunization include vaccines for adults with eligibility depending on indigenous status, age, and presence of risk factors [115,116]. The Australian NIP recommends several vaccines for adults (Table 1), including annual vaccination against influenza, and COVID-19 and vaccination against HZ and pneumococcal disease for all indigenous people (Aboriginal and Torres Strait Islanders) aged ≥50 years [115]. Recommendations for non-indigenous adults aged ≥65 years include vaccination against HZ, and for those aged ≥70 years, vaccination against pneumococcal disease. Vaccination against influenza, RSV, and pertussis is recommended for pregnant women. Additional recommendations for high-risk groups in Australia include vaccination against influenza, Hib, HZ, and meningococcal and pneumococcal diseases. The Australian NIP provides funding of vaccines for eligible high-risk groups. For indigenous adults, pneumococcal vaccination is funded for adults ≥ 50 years [115,117].

Compared to high-income countries, adult vaccination programs in low- and middle-income countries face limited financial allocation, failed implementation of already adopted NIPs, a scarce number of targeted VPDs, and less frequent updates [118,119]. In addition, the infrastructure and delivery mechanisms for reaching adults are not as robust. Despite many obstacles, over the past 20 years, revolutionary global progress has been achieved in adult immunization, with significant differences between and within countries [119]. Sharing knowledge and best practices in adult immunization, along with public awareness raising and vaccine policy advocacy by public health institutions, is essential to improving adult vaccination strategies worldwide.

## 12. Why Do National Vaccination Programs Differ?

Vaccination programs vary significantly both within and between countries [5,85,120]. There are significant differences in vaccination recommendations according to age and target groups, the number and type of vaccines included, and the vaccination schedule [5,85]. The selection of vaccines for inclusion in an immunization program is primarily based on the available evidence regarding their safety, efficacy, and effectiveness [120]. In addition, the vaccination program is shaped by the burden of VPDs and the goals of disease elimination and control [121]. However, numerous other non-medical influences such as political and economic factors, media campaigns, and antivaccine movements can strongly influence vaccination policies [121].

Each NIP is unique and reflects the economic capacity, healthcare system capacity, and political will [122]. In some countries, vaccination is voluntary, in others it is mandatory, while in a number of countries both approaches are practiced. The introduction of mandatory immunization even in countries that have a long history of voluntary vaccinations has been triggered by outbreaks of VPDs, low vaccination coverage, and increasing parental refusal to vaccinate children [5,123]. Mandatory vaccination may be an option if confidence in the safety and efficacy of vaccines is high and the expected benefits outweigh alternative strategies [124].

The design of vaccination programs is fraught with challenges in low-income countries, due to low financial resources and inadequate capacities for vaccine implementation [122,125]. Currently, there is no globally acceptable consensus-based vaccination program despite clear evidence of the benefits of vaccination [5,121,126]. Regardless of the differences, effective NIPs require public trust, strong healthcare systems, and adaptable vaccination strategies [127].

## 13. Development of New Vaccines Against Old and Emerging Pathogens and Public Health Threats

Vaccines are needed for several emerging and re-emerging pathogens. Human metapneumovirus (hMPV) is a re-emerging respiratory virus that causes significant morbidity and mortality, particularly among young children, immunocompromised individuals, and older adults [128]. Currently, an mRNA vaccine targeting fusion proteins of both hMPV and parainfluenza virus type 3 (PIV3) is under clinical assessment [129]. Preliminary results indicate a good safety and tolerability profile and a notable increase in neutralizing antibody titers against hMPV and PIV3 after a single dose [129]. Overall, the mRNA vaccine technology has greatly advanced the landscape of vaccinology in the past few years through the rapid development of vaccines with a satisfactory safety profile and low productivity costs [130]. Currently, many mRNA vaccines are under development or in clinical trials, e.g., against seasonal and avian influenza, hMPV, cytomegalovirus, VZV, Epstein–Barr virus (EBV), tuberculosis, chikungunya virus, and *Plasmodium falciparum* [130].

Antimicrobial resistance is a major public health problem globally. The development of vaccines against multidrug-resistant pathogens is expected to reduce the burden of antimicrobial resistance and healthcare-associated infections. Currently, vaccines against *Clostridium difficile*, *Staphylococcus aureus*, and Gram-negative bacteria are under development or in clinical trials [131]. Nevertheless, the identification of risk groups who may benefit from these vaccines might prove challenging and complex [131].

Lastly, as life expectancy has been extended, therapeutic vaccines targeting chronic infectious diseases with an increased medical, societal, and economic burden are needed. Therapeutic vaccines for chronic infectious diseases work through stimulation of the immune system and blockage of the development of diseases caused by persistent infection [132]. Relevant clinical research is still undergoing while standards to assess the efficacy of therapeutic vaccines are lacking [132]. Currently there are therapeutic vaccines against human papillomavirus and EBV-related tumors under investigation [132].

Beyond the development of new, safe, and effective vaccines against old and emerging pathogens, achieving high vaccine coverage rates is equally important. In recent years, community pharmacies have been successfully involved in the delivery of vaccinations to adults [133,134]. Nevertheless, vaccine hesitancy and disparities in vaccine accessibility were critical barriers in vaccination campaigns during the COVID-19 pandemic globally. These obstacles should be addressed to maximize vaccination benefits for all in the coming years but also to successfully confront future pandemics.

## Figures and Tables

**Table 1:** Vaccination programs for adults in 30 European countries, United States, and Australia *.

Country	COVID-19	Diphtheria	Tetanus	Pertussis	HiB	Hep A	Hep B	HZ	HPV	Influenza	Measles	Mumps	Rubella	Men	Polio	TBE	Varicella	Pneumo	RSV
EU/EEA [12]																			
Austria																			
Belgium																			
Bulgaria																			
Croatia																			
Cyprus																			
Czechia																			
Denmark																			
Estonia																			
Finland																			
France																			
Germany																			
Greece																			
Hungary																			
Iceland																			
Ireland																			
Italy																			
Latvia																			
Liechtenstein																			
Lithuania																			
Luxembourg																			
Malta																			
Netherlands																			
Norway																			
Poland																			
Portugal																			
Romania																			
Slovakia																			
Slovenia																			
Spain																			
Sweden																			
United States [113,114]																			
Australia [115]																			

COVID-19: coronavirus disease 2019; Hib: *Haemophilus influenzae* type b; HepA: hepatitis A; HepB: hepatitis B; HZ: herpes zoster; HPV: human papillomavirus; Men: meningococcal disease; Pneumo: pneumococcal disease; Polio: poliomyelitis; RSV: respiratory syncytial virus; TBE: tick-borne encephalitis. * not including recommendations for vaccination against rabies and monkeypox.

## Data Availability

Not applicable.
